# Future climate effects on suitability for growth of oil palms in Malaysia and Indonesia

**DOI:** 10.1038/srep14457

**Published:** 2015-09-24

**Authors:** R. Russell M. Paterson, Lalit Kumar, Subhashni Taylor, Nelson Lima

**Affiliations:** 1 CEB - Centre of Biological Engineering, University of Minho, 4710-057 Braga, Portugal; 2 School of Environmental and Rural Science, University of New England, Armidale NSW 2351, Australia; 3 Postgraduate Program in Agricultural Microbiology, Federal University of Lavras, 37200-000, Lavras, MG, Brazil

## Abstract

The production of palm oil (PO) is highly profitable. The economies of the principal producers, Malaysia and Indonesia, and others, benefit considerably. Climate change (CC) will most likely have an impact on the distribution of oil palms (OP) (*Elaeis guineensis*). Here we present modelled CC projections with respect to the suitability of growing OP, in Malaysia and Indonesia. A process-oriented niche model of OP was developed using CLIMEX to estimate its potential distribution under current and future climate scenarios. Two Global Climate Models (GCMs), CSIRO-Mk3.0 and MIROC-H, were used to explore the impacts of CC under the A1B and A2 scenarios for 2030, 2070 and 2100. Decreases in climatic suitability for OP in the region were gradual by 2030 but became more pronounced by 2100. These projections imply that OP growth will be affected severely by CC, with obvious implications to the economies of (a) Indonesia and Malaysia and (b) the PO industry, but with potential benefits towards reducing CC. A possible remedial action is to concentrate research on development of new varieties of OP that are less vulnerable to CC.

Climate plays an important role in defining the range limits of species’ distributions by exerting eco-physiological constraints^1^. However, factors such as soil properties and biotic interactions may prevent species from colonizing sites that are otherwise suitable^2^. The interrelationships among the various factors that influence distributions can be complex since the spatial scale at which each factor acts can vary widely^3^. Climate acts at a broad scale to limit species’ distributions whilst at local scales other factors may become progressively more important, resulting in a mixture of occupied and unoccupied sites within climatically suitable areas^4^. Topography, soil texture and nutrient content may become significant at finer, local scales. Changes in climate will, therefore, have broad-scale impacts on the distribution of agricultural species such as oil palm (OP). Such changes can be investigated using ecological niche modelling approaches; however, the impacts of non-climatic factors are not captured by these modelling methodologies, although these factors can be considered in a stepwise manner after the climate modelling has been completed using a Geographical Information System (GIS).

OP is one of the world’s most rapidly expanding crops and the primary source of vegetable oil and fat. Rural development, economic stimulation, and reduced poverty result from the palm oil (PO) industry in many equatorial countries^5^. The oil is used (a) in c. 30% of foods, pharmaceuticals and cosmetics, (b) for cooking and (c) as biodiesel for motorized vehicles^6^. Malaysia’s rapid economic development corresponds with large scale OP cultivation and the country was the second largest producer of PO in 2008 at 83 million tonnes, with Indonesia producing 85 million tonnes. OP cultivation in Malaysia increased from 5.4 × 10^4^ to 4.7 × 10^6^ ha from 1960 to 2009. Production of crude PO increased from 9.4 × 10^4^ to 1.8 × 10^7^ tonnes from 1960 to 2009 and exports increased from 2.17 × 10^7^ to 2.24 × 10^7^ tonnes from 2008 to 2009^6^. Furthermore, Indonesian PO production generated US$11.1 billion in 2010 and Indonesia plans to double production primarily by expanding holdings in Kalimantan and Papua^7^.

Surface temperature in most areas of Malaysia has increased over the last four decades at rates of 2.7–4.0 ^o^C per 100 years^8^. However, some areas in southwestern Borneo revealed lower or insignificant warming trends. Long-term upward trends in precipitation since the mid-1970s are also apparent in some regions. Most determinations from the west coast of Peninsular Malaysia show increases of annual and seasonal rainfall during northeast and southwest monsoons since the mid-1970s related to the increasing trends of maximum daily rainfall. The maximum length of dry spells also increased during this period^8^. The mean surface temperature of Malaysia increased from 0.6 to 1.2 °C during 1969–2009 and was projected to increase by 1.5 to 2.0 °C by 2050. Rainfall and river flows may experience greater fluctuations^9^. Projections indicate that maximum monthly precipitation will increase by up to 51% in Pahang, Kelantan and Terengganu, and the minimum precipitation will decrease by 32 to 61% for Peninsular Malaysia. Annual rainfall will increase by 10% in Kelantan, Pahang, Terengganu, and the north-west Coast and decrease by 5% in Johor and Selangor causing risk and uncertainty^10^ for OP cultivation. Nevertheless, more objective data on the effects of climate change (CC) on OP are required.

Projections concerning how crops will be affected by CC are numerous^11^, but high quality information on the impacts of CC on OP is lacking^6^. For example, the crop is only mentioned once in a comprehensive review of the impacts of CC on tropical crops^12^. Past modelling studies have focused on the economic aspects of OP cultivation in Malaysia and Indonesia^13^, the carbon accumulation rates of OP plantations^14^, impacts of OP plantations on land use changes in Brazil^15^and the impact of emissions from OP plantations on air quality and climate^16^. Furthermore, a modelling study in Malaysia has investigated the carbon sequestration and greenhouse gas emissions associated with OP cultivation and land-use change within the country^17^. However, none of these have explicitly investigated the impacts of CC on the potential distribution of OP.

Tropical plants are often at the limits of growth, where small detrimental changes in climate can affect survival^6^. In general, more crops and greater yields are projected to occur in regions that are currently cool (e.g. sub-tropical) while fewer crops and yields are projected to occur in regions that are currently hot (e.g. tropical)^18^. These projections will have implications for OP production which is strongly affected by CC and the increasing frequency of climate anomalies. Research indicates that OP yield will be reduced by CC in many areas of Malaysia, making its economic viability difficult^10^. For example, the increasing frequency of drought in SE Asia has caused declines of 10–30% in PO production while estimated reduction in crude PO production caused by CC in southern Malaysia was 26.3%^19^. Furthermore, yields are projected to decrease by approximately 30% should temperature increase 2 °C above optimum and rainfall decrease by 10%^20^. A combination of general circulation models and economic information indicated that a temperature variation of 0.6 to 1.4 °C and ± 15% rainfall variation led to a positive change in earnings for PO of up to $2,453 yr^−1^ while earnings were reduced to $1181 yr^−1^ with ± 32% rainfall fluctuation and moderate temperature fluctuation^19^. Consequently, the countries which currently cultivate OP will face increasing uncertainty in the future.

Different opinions exist regarding the future impact on PO production^21^ and this current study attempts to clarify some of the uncertainties associated with the impacts of CC on OP cultivation. To this end, we utilized the CLIMEX modelling package to develop a model of the climate responses of OP. This model was then used to project its potential distribution under current and future climate for Malaysia and Indonesia for 2030, 2070 and 2100. It is important to note that CLIMEX is climate-based and does not cover other biophysical factors such as soils, vegetation cover and disturbance activities. In the case of agricultural crop distributions, human inputs such as improved pest and weed management will also impact where a crop will grow; however, these aspects are not included in CLIMEX modelling.

## Results

### Current climate to 2030

**Figure 1:**
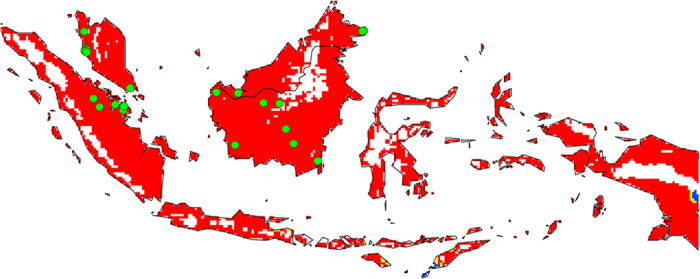
The climate (EI) for *Elaeis guineensis* based on CLIMEX for reference climate (averaging period 1950–2000). White areas indicate unsuitable climate areas (EI = 0), blue areas indicate marginal climate areas (EI = 1–10), yellow areas indicate suitable climate areas (EI = 10–20) and red areas indicate highly suitable climate areas (EI > 20). Data for current Malaysian and Indonesian distribution (green dots) are taken from the Global Biodiversity Information Facility^52^ (GBIF, 2010) and literature. The CLIMEX results were exported into GIS software (ArcGIS Software Version 10.2. Environment Systems Research Institute, Redlands, CA) to generate the map in this figure.

**Figure 2:**
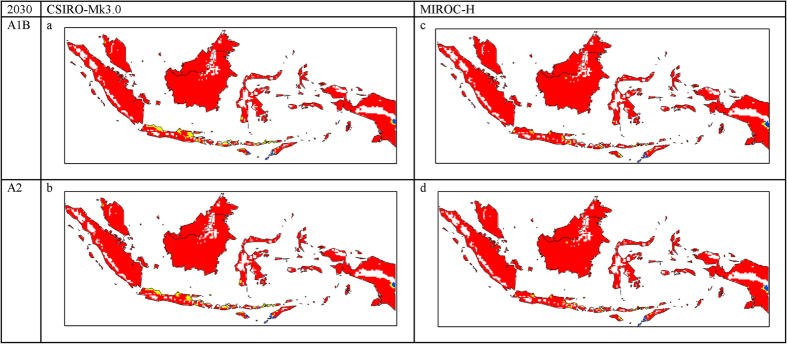
The climate (EI) for *E. guineensis* based on CLIMEX for 2030 under the: (a). CSIRO-Mk3.0 global climate model running the SRES A1B; (**b**) CSIRO-Mk3.0 global climate model running the SRES A2; (**c**) MIROC-H global climate model running the SRES A1B and (**d**) MIROC-H global climate model running the SRES A2. White areas indicate unsuitable climate areas (EI = 0), blue areas indicate marginal climate areas (EI = 1–10), yellow areas indicate suitable climate areas (EI = 10–20) and red areas indicate highly suitable climate areas (EI > 20). The CLIMEX results were exported into GIS software (ArcGIS Software Version 10.2. Environment Systems Research Institute, Redlands, CA) to generate the map in this figure.

The final map resulting from the modelled current distribution was validated using the occurrence data from SE Asia (Fig. 1). Since these locations were not used for model development, they provided independent validation. All of the occurrence points within Malaysia and Indonesia, a total of 23, fall within highly suitable climatic areas for OP. Furthermore, our modelled distribution shows a good match to OP plantations in SE Asia as described in the literature^22^,^23^. Changes in the area of climatic suitability for OP cultivation are shown in Table 1. The validation indicates that the present SE Asian distribution of OP is consistent with the Ecoclimatic Index values resulting from the CLIMEX model. The results show that approximately 1.8 million km^2^ of the land area in this region has a suitable to highly suitable climate for OP with 0.339 million km^2^ being marginal to unsuitable. There are no substantial differences in the projections for 2030 (Fig. 2a to d) when compared to the results for current climate. However, a change from highly suitable to suitable climate is projected, especially in the north coast of Java and this change is more pronounced with the CSIRO-Mk3.0 Global Climate Model (GCM) (Fig. 2a,b). There is little change in climatic suitability for OP production in Papua by 2030.

### 2070

**Figure 3:**
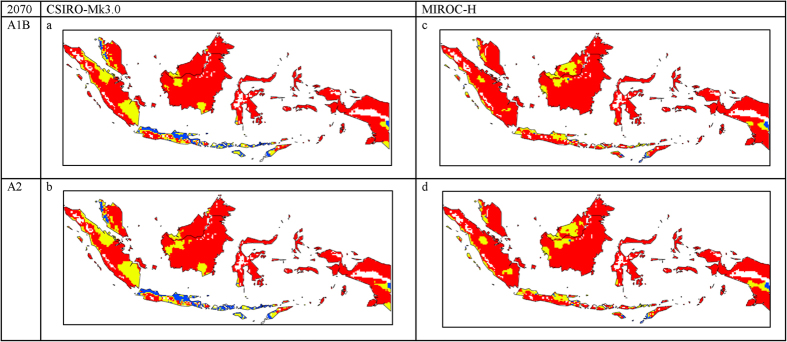
The climate (EI) for *E. guineensis* based on CLIMEX for 2070 under the: (a). CSIRO-Mk3.0 global climate model running the SRES A1B, (**b**) CSIRO-Mk3.0 global climate model running the SRES A2; (**c**) MIROC-H global climate model running the SRES A1B and (**d**) MIROC-H global climate model running the SRES A2. White areas indicate unsuitable climate areas (EI = 0), blue areas indicate marginal climate areas (EI = 1–10), yellow areas indicate suitable climate areas (EI = 10–20) and red areas indicate highly suitable climate areas (EI > 20). The CLIMEX results were exported into GIS software (ArcGIS Software Version 10.2. Environment Systems Research Institute, Redlands, CA) to generate the map in this figure.

A change in climatic suitability from highly suitable (1.79 million km^2^ under current climate to ranging between 1.56 to 1.77 million km^2^ by 2070) to merely suitable (0.008 million km^2^ under current climate to ranging between 0.21 and 0.34 million km^2^ by 2070) is projected for Malaysia and Indonesia (Fig. 3a to d and Table 1). The projections under CSIRO-Mk3.0 GCM show a more pronounced reduction in climatic suitability, changing from highly suitable under current climate to marginal by 2070 for Java and the islands, and western peninsular Malaysia (Fig. 3a,b). The climate in Timor is projected to become increasingly less suitable for OP production. A change from highly suitable to merely suitable climate by 2070 can be seen for some parts of Papua and this change is more pronounced under the MIROC-H GCM (Fig. 3c,d).

### 2100

**Figure 4:**
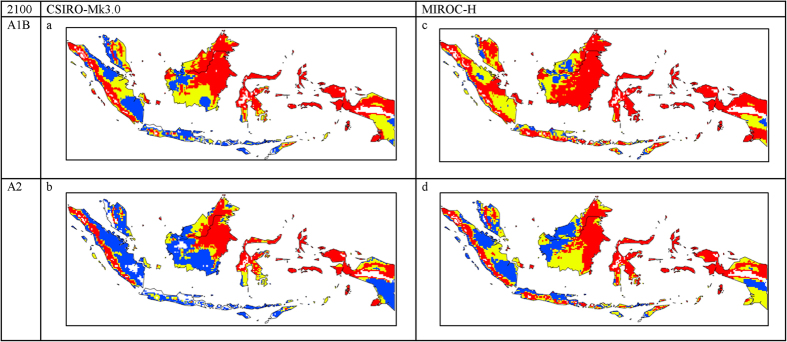
The climate (Ecoclimatic index (EI) for *E. guineensis* based on CLIMEX for 2100 under the: (a). CSIRO-Mk3.0 global climate model running the SRES A1B; (**b**) CSIRO-Mk3.0 global climate model running the SRES A2; (**c**) MIROC-H global climate model running the SRES A1B and (**d**) MIROC-H global climate model running the SRES A2. White areas indicate unsuitable climate areas (EI = 0), blue areas indicate marginal climate areas (EI = 1–10), yellow areas indicate suitable climate areas (EI = 10–20) and red areas indicate highly suitable climate areas (EI > 20). The CLIMEX results were exported into GIS software (ArcGIS Software Version 10.2. Environment Systems Research Institute, Redlands, CA) to generate the map in this figure.

By 2100 the situation is markedly less favorable for the two countries (Fig. 4a to d and Table 1). Large areas of marginal climate, ranging from 0.139 to 0.821 million km^2^, cover the regions and small areas of unsuitable climate become apparent in Indonesia, particularly under the CSIRO-Mk3.0 GCM with the A2 scenario (Fig. 4b). The projection under the MIROC-H GCM with the A1B scenario does not indicate such a large change, although there is a definite reduction in climatic suitability (Fig. 4c). There is a pronounced reduction in climatic suitability by 2100 in Papua and this situation is mirrored predominantly in Kalimantan. These projections have major implications for current plans to expand OP plantations in these areas.

**Figure 5:**
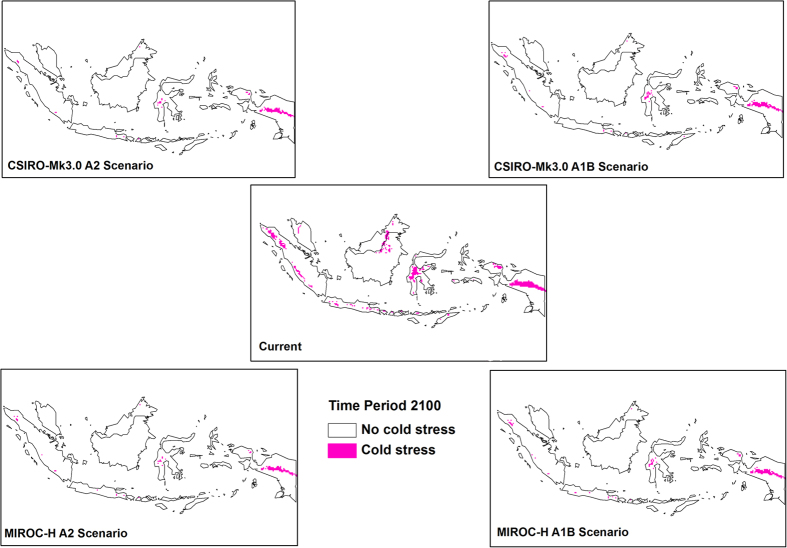
Changes in cold stress from historical climate to 2100. The CLIMEX results were exported into GIS software (ArcGIS Software Version 10.2. Environment Systems Research Institute, Redlands, CA) to generate the map in this figure.

**Figure 6:**
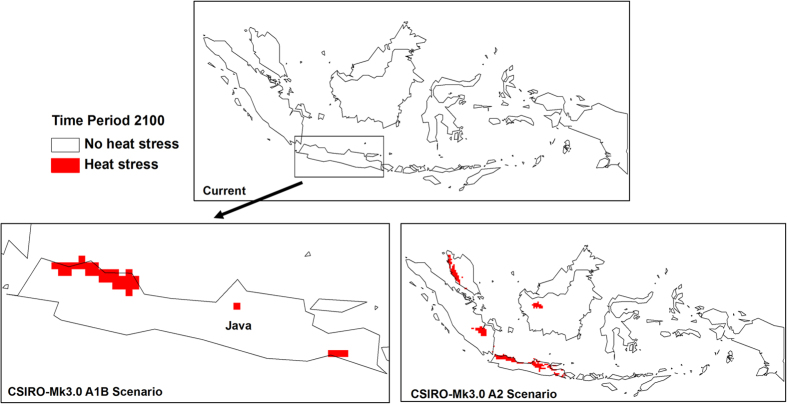
Changes in heat stress from historical climate to 2100. The CLIMEX results were exported into GIS software (ArcGIS Software Version 10.2. Environment Systems Research Institute, Redlands, CA) to generate the map in this figure.

**Figure 7:**
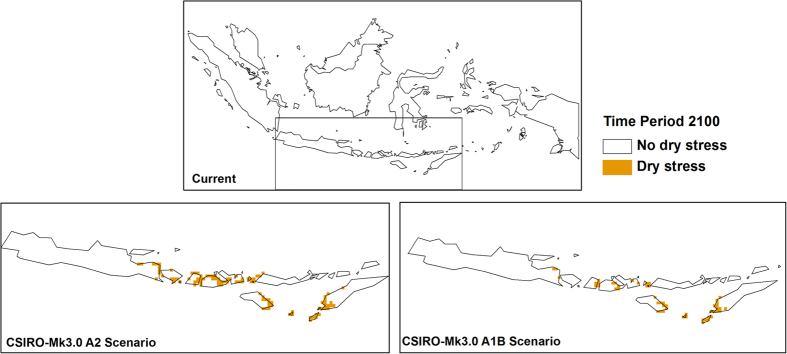
Changes in dry stress from historical climate to 2100. The CLIMEX results were exported into GIS software (ArcGIS Software Version 10.2. Environment Systems Research Institute, Redlands, CA) to generate the map in this figure.

Cold stress played an important role in the changes in climatic suitability for OP in Malaysia and Indonesia (Fig. 5). A reduction in cold stress can be observed by the end of the century in Sumatra, Java, West Papua, Sulawesi and Borneo. Conversely, an increase in heat stress (Fig. 6) and dry stress (Fig. 7) can be seen in some parts of the region and these are more pronounced under the CSIRO-Mk3.0 GCM.

## Discussion

The results indicate a reduction in climatic suitability under future climate for OP production in Malaysia and Indonesia. Decreases in suitability are gradual by 2030, but become more pronounced by 2100. These projections imply that PO production will be severely affected by CC, with obvious implications for the economies of Indonesia and Malaysia and for the international manufacture of PO products. Changes in cold, heat and dry stresses were largely responsible for the changes in climatic suitability for OP cultivation while no substantial variations in wet stress were observed. It must be noted that the climate suitability projections reported herein are potential distributions and not predicted future distributions. CLIMEX is based only on climate. Non-climatic factors that affect species’ distributions, such as biotic interactions, soil type and topography are not included explicitly in the modelling process. The other factors may cause the actual range of this species to fall below the potential. Furthermore, there are uncertainties associated with (a) the state of climate modelling and (b) future global greenhouse gas emission patterns. This means that models based on future climate scenarios, such as in this study, should be treated as elaborate sensitivity analyses which indicate the direction and magnitude of change that may be expected in the future.

The projected temperatures in Malaysia and Indonesia from CC will be extremely high^18^ and may lead to the inability to grow OP in some areas. However, the growth of OP might become optimal in currently subtropical regions^6^ as a consequence of the general movement of crops to the Poles^24^. There are three basic scenarios likely to occur worldwide: Climate will (a) remain suitable, although the crop may experience more stress such as disease (see below); (b) become unsuitable for OP and the crop may experience periods of increasing stress also leading to disease; and (c) become suitable for OP providing an opportunity to grow disease-free palms^6^.

The slow growth rate of OP when compared to perennial crops, suggests that currently planted crops will experience the effects of CC in the future as they grow to full maturity^9^. Replanting will reflect this long cycle compared to other crops such as maize. Changes in climate will have implications for modified OP which may be bred in the long term to survive CC and increased threats of diseases^25^.

Other factors affect OP growth, yields and survival apart from climate *per se* such as pests and diseases from (a) decreased resistance of OP, and (b) the development of novel pest and diseases (e.g. insects, bacteria and fungi). CC will also affect plant diseases, thus adding to the uncertainty of OP cultivation in the future^26^,^27^,^28^, although CC may actually decrease the severity of epidemics in some cases^29^. As suitable climates for OP cultivation change, the current sub-tropical countries consisting of the major developed countries, e.g. parts of the USA, may manage disease more effectively from better technologies^30^.

The temperatures resulting from CC may be too high for the current diseases of OP to continue being infective^6^ and changes may occur in the type, amount and importance of pathogens and diseases. Importantly, host resistance may be overcome more rapidly due to accelerated pathogen evolution from increased fecundity at high CO_2_ and/or enhanced UV-B radiation. Global warming will favour the emergence of new diseases, because the (i) distributional range, temporal activity and community structure of pathogens will be modified^26^ and (ii) phenology and conditions of the hosts will be altered^31^,^32^. Modelling studies have investigated the effects of CC on the distribution of diseases of tropical crops, although little empirical knowledge exists for the development of adaptation strategies^12^. Deutsch *et al.*^33^ observed that warming in the tropics is likely to have the most deleterious consequences because tropical insects are sensitive to temperature change and are currently living very close to their optimal temperature. The lack of vectors will make some diseases less likely, although diseases such as *Ganoderma* rots of OP are not dependent on spread by insects^34^. Paterson *et al.*^6^ provide details of the effect of CC on diseases of other tropical crops relevant to OP. However, long-term datasets are rare in relation to tropical and plantation crop diseases, which are a prerequisite for detecting fingerprints of inter-annual climatic variation on plant diseases^35^.

The most probable impact of elevated CO_2_ on OP disease epidemics would be from changes in host physiology and morphology^29^, rather than a more infective pathogen (e.g. better penetration)^36^. Emissions of CO_2_ and SO_2_ from CC and pollution are affecting plant/pathogen interactions in natural and agricultural ecosystems worldwide^26^, and will be relevant to OP disease. The ability of OP disease fungi to mutate and respond to opportunities arising from change is a key factor in considering the potential impact of CC^6^. CC may affect not only the geographical range and abundance of vectors, but also the interaction between a pathogen and a vector (e.g. the pathogen may be transmitted by novel vectors). An important indirect factor is that the feeding rate of many arthropod vectors increases at higher temperatures, thus increasing exposure of crops to OP disease^37^.

The human sway on the climate system is transparent, and anthropogenic emissions of greenhouse gases are now the highest ever^38^. Most of the global warming during the next 30 years will be due to emissions that have already occurred. Over the longer term, the degree and pace of warming mainly depend on current and near future emissions^39^. CC is caused to a large extent by emissions of CO_2_; however, forests are a sink for CO_2_ and Malaysia and Indonesia have extensive tropical forests. Indonesia had the highest rate of forest cover loss from 2000 to 2012^5^ from conversion to, amongst other crops, OP plantations^40^. In addition, the peat reserves in SE Asia have an even greater capacity to retain CO_2_ but are cleared for OP plantations. Forest destruction has a large climate impact, especially for those on peatlands^41^, where CO_2_ is released into the atmosphere and contributes to global warming when these two resources are burned^40^. Indonesia is the third largest global emitter of CO_2_ because of this and the high carbon stocks in above-ground and subterranean pools^5^,^42^. Interestingly, mineral soils received 87% of plantation development and generated c. 70% of gross emissions from Kalimantan OP from 1990 to 2010^7^. A consequence of the reduced ability of Malaysia and Indonesia to grow OP as described herein, will be a decrease in the contribution to CC through deforestation to create more OP plantations. However, a reduction in deforestation will only occur if there is no corresponding increase (a) in other crops more adapted to CC, and/or (b) in other activities such as more logging to compensate for reduced OP growth. Perennial crops would be less affected by CC in the short term because of the shorter crop cycle and continued deforestation could still occur to grow increasing amounts of these crops, at least in the short to medium term. However, current practices for producing PO in Malaysia and Indonesia may lead to a substantially reduced capacity for production in the long term.

Mitigation has gained prominence as an important response to CC in vulnerable countries, although it is clear that some impacts are now unavoidable. Countries have applied various approaches and techniques to mitigate the impacts of climatic vulnerabilities including Malaysia^10^, which is considering reducing emissions from deforestation and forest degradation, together with forest conservation, sustainable management and enhancement of forest carbon stocks (REDD+)^43^. Malaysia has employed eight entry point projects (EPP), such as replanting low yielding palms, as alternatives to increasing the size of plantations, although the question remains whether the REDD+ programme would generate sufficient financial incentives to compete with the EPP^43^. Improving the efficiency of current OP production practices also reduces pressure from CC. Additionally, the Malaysian Palm Oil Board has advocated demonstrating the sustainability of OP products^44^ in relation to CC, often by undertaking life cycle assessments of products such as PO^45^. Finally, plant breeding programmes^25^could be intensified to develop varieties that are less impacted by CC.

## Methods

### CLIMEX Software

The CLIMEX software has been used previously to assess the impacts of CC on agricultural productivity^46^. The potential distribution model of OP under current and future climate scenarios was developed using CLIMEX for Windows Version 3^47^ (Hearne Scientific Software Pty Ltd, Melbourne 2007). This software is based on the observation that the distribution of plants and poikilothermal animals is primarily determined by climate^48^. An eco-physiological model forms the basis of the software and works on the assumption that at each location, a species may experience a favourable season with positive population growth and an unfavourable season that causes population decline^47^. The model parameters that describe the species’ response to climate can be inferred by the user based on its geographic range or phenological observations^47^. A deductive approach can also be taken to apply climate response parameters extracted from experimental observations to climatic datasets. In practice, both approaches can be applied to inform the selection of parameter values. The fitted parameters can then be applied to novel climates to project the species’ potential range in new regions or climate scenarios^49^,^50^. An annual growth index (GIA) is used to describe the potential for population growth during favourable climate conditions while stress indices (cold, wet, hot and dry) and interaction stresses (hot-dry, hot-wet, cold-dry and cold-wet) describe the probability that the population can survive unfavourable conditions. The growth and stress indices are calculated weekly and combined into an overall annual index of climatic suitability, the Ecoclimatic index (EI) which is theoretically scaled from 0 to 100. Establishment is only possible if EI > 0; 1–10 indicates marginal habitats, 10–20 can support substantial populations while >20 are highly favourable^51^, and a detailed description of parameters can be found in this reference.

### Climate Data and Climate Change Scenarios

The modelling was carried out using the CliMond 10´gridded climate data^52^. Average minimum monthly temperature (T_min_), average maximum monthly temperature (T_max_), average monthly precipitation (P_total_) and relative humidity at 09:00 h (RH_09:00_) and 15:00 h (RH_15:00_) were used to represent historical climate (averaging period 1961–1990). The potential future climate in 2030, 2070 and 2100 were characterized using the same five variables based on two Global Climate Models (GCMs), CSIRO-Mk3.0^53^ and MIROC-H (Centre for Climate Research, Japan) with the A1B and A2 SRES scenarios^18^. These were available as part of the CliMond dataset. The two GCMs were selected from 23 GCMs for the CliMond dataset based on three criteria^52^.

1. The temperature, precipitation, mean sea level pressure and specific humidity variables required for CLIMEX were available for these two GCMs.
2. The models have relatively small horizontal grid spacing.
3. They performed well compared to other GCMs in representing basic aspects of observed climate at a regional scale^54^.

The A1B and A2 scenarios were selected to typify the range of possible climate suitability for OP in 2030, 2070 and 2100. The A1B scenario portrays a balance between the use of fossil and non-fossil resources. On the other hand, the A2 describes a varied world with high population growth but slow economic development and technological change. No scenarios from the B family of SRES scenarios were included in this because of the observation that some parameters such as global temperature and sea level rise are presently increasing at a much greater rate than predicted by the hottest B family of SRES scenarios^55^. The future projection years of 2030, 2070 and 2100 were selected because they provide a reasonable snapshot of three time periods; one in the near future in 15 years’ time, one in the mid-term in 55 years’ time and one much later in the future in 85 years’ time.

### Fitting CLIMEX Parameters

The Global Biodiversity Information Facility^56^ is a database of natural history collections around the world for various species and is available for download. Information on the global distribution of OP was downloaded (Fig. 1) and used in parameter fitting. Even though the primary aim of the study was to investigate the impacts of CC on OP distribution in Malaysia and Indonesia, the *global* occurrence of OP was used to inform the parameter fitting process. This process aims to capture the “climatic envelope” of the species, i.e. the climatic conditions under which this species survives throughout the world. This ensures that the parameters reflect the climate of all the regions of the world where OP currently occurs. A total of 398 records were downloaded but many did not have geographic coordinates or were repetitions and such records were removed, leaving 85 records. A further 39 records were found through a literature search making a final total of 124 records (Fig. 1). Stress parameters were fitted using the known native distribution in Africa and the naturalized distribution in South America. Phenology data from literature were used to assist in fitting the growth parameters^9^,^57^,^58^,^59^,^60^,^61^,^62^. Each of the parameters was adjusted iteratively until a satisfactory agreement was reached between the potential and known distribution of OP in these areas. The parameters were checked to ensure that they were biologically reasonable (Table 2). South East Asian distribution data was not used in model development and reserved for validation of the model.

### Cold Stress

The northernmost occurrences of OP in Africa have been reported in Guinea, between 10-11 °N^57^ while in Central and South America OP is mainly grown in the humid tropics stretching from 19 °N in Dominican Republic to 15 °S in Brazil^58^. Thus, two cold stress mechanisms were used to define the southern and northern limits of OP distributions in Africa and South America. The growth of OP seedlings is totally inhibited below 15 °C under controlled conditions^62^. Therefore, the cold stress temperature threshold (TTCS) was set at 15 °C with the stress accumulation rate (THCS) set at −0.005 week^−1^. The Cold-Stress Degree-day Threshold (DTCS) was set at 20 °C days, with the stress accumulation rate (DHCS) set at −0.0005 week^−1^. These two mechanisms ensured that the potential distribution was restricted to the known southern limits in Brazil and northern limits in Guinea.

### Heat Stress

In Nigeria, there are no semi wild palms north of 7 °N except in particularly favored areas where there are shallow water tables^57^. The heat stress parameter (TTHS) was set at 36 °C, the same level as the limiting high temperature (DV3) with a stress accumulation rate (THHS) of 0.001 week^−1^, which allowed OP to persist at about 7 °N in Nigeria.

### Dry Stress

The dry stress parameter was set at the same level (0.4) as the lower soil moisture threshold (SM0) because soil moisture related stresses begin at the same soil moisture levels where growth stops. The stress accumulation rate of −0.007 week^−1^ was set to exclude the species from eastern Africa as it is unsuitable for OP because it is too dry^59^.

### Wet Stress

The wet stress threshold (SMWS) was set to 2 and the accumulation rate (HWS) set at 0.0023 week^−1^ since the ideal rainfall requirements of OP are 2000 to 2500 mm annually^59^.

### Temperature Index

The ideal mean maximum temperature for OP is between 29 °C to 33 °C while the ideal mean minimum temperature is between 22 °C to 24 °C^59^. Temperatures below 20 °C and above 36 °C are unsuitable for growth^59^. Thus, the limiting low temperature (DV0) was set at 19 °C, the lower (DV1) and upper (DV2) optimal temperatures were set at 24 °C and 28 °C, respectively. The limiting high temperature (DV3) was set at 36 °C. These provided a good fit to the observed global distribution.

### Moisture Index

Rainfall levels of 2000 to 2500 mm per year have been reported as highly suitable for OP, however, it will tolerate rainfall levels of up to 3000 mm per year^59^. Thus, the lower moisture threshold (SM0) was set at 0.4, the lower (SM1) and upper (SM2) optimal soil moisture were set at 0.6 and 1.6, respectively and the limiting soil moisture (SM3) was set at 2. These provided a good match to the observed global distributions.

### Degree day threshold

The length of the growing season can also limit the distribution of species and this is described by the degree day threshold parameter. In Madagascar, OP is reported to occur as far south as 21 °S^59^. The threshold minimum heat accumulation parameter (PDD) was adjusted to allow the species to occur at the South-Eastern climate stations in Madagascar.

## Additional Information

**How to cite this article**: Paterson, R. R. M. *et al.* Future climate effects on suitability for growth of oil palms in Malaysia and Indonesia. *Sci. Rep.* **5**, 14457; doi: 10.1038/srep14457 (2015).

## Figures and Tables

**Table 1:** Changes in areas of climatic suitability for *Elaeis guineensis* cultivation under different scenarios for Indonesia and Malaysia.

Scenario	Area (km^2^)			
	Unsuitable	Marginal	Suitable	Highly Suitable
Current	332,880	6,122	7,908	1,794,493
2030 A1B CS	220,135	13,264	49,741	1,858,263
2030 A2 CS	228,807	11,479	44,639	1,856,478
2030 A1B MR	225,236	8,928	22,192	1,885,047
2030 A2 MR	235,184	8,673	19,896	1,877,649
2070 A1B CS	139,784	86,983	291,047	1,623,589
2070 A2 CS	136,723	100,502	344,104	1,560,074
2070 A1B MR	143,355	17,856	208,911	1,771,281
2070 A2 MR	136,213	21,427	252,785	1,730,978
2100 A1B CS	123,969	415,782	593,573	100,8079
2100 A2 CS	202,279	821,360	387,723	730,041
2100 A1B MR	104,583	139,274	519,600	1,377,946
2100 A2 MR	83,922	526,232	630,815	900,435

**Table 2:** CLIMEX parameter values used for *Elaeis guineensis.*

Parameter	Mnemonic	Values
Limiting low temperature	DV0	19 °C
Lower optimal temperature	DV1	24 °C
Upper optimal temperature	DV2	28 °C
Limiting high temperature	DV3	36 °C
Limiting low soil moisture	SM0	0.4
Lower optimal soil moisture	SM1	0.6
Upper optimal soil moisture	SM2	1.6
Limiting high soil moisture	SM3	2
Cold stress temperature threshold	TTCS	15 °C
Cold stress temperature rate	THCS	−0.005 week^−1^
Minimum degree-day cold stress threshold	DTCS	20 °C days
Degree-day cold stress rate	DHCS	−0.0005 week^−1^
Heat stress temperature threshold	TTHS	36 °C
Heat stress temperature rate	THHS	0.001 week^−1^
Dry stress threshold	SMDS	0.4
Dry stress rate	HDS	−0.007 week^−1^
Wet stress threshold	SMWS	2
Wet stress rate	HWS	0.0023 week^−1^
Degree-day threshold	PDD	1500
